# Accuracy of gross intraoperative margin assessment for breast cancer: experience since the SSO-ASTRO margin consensus guidelines

**DOI:** 10.1038/s41598-020-74373-6

**Published:** 2020-10-15

**Authors:** Alberto Nunez, Veronica Jones, Katherine Schulz-Costello, Daniel Schmolze

**Affiliations:** 1 Beckman Research Institute, City of Hope National Medical Center, 1500 East Duarte Road, Duarte, CA 91010 USA; 2 Department of Surgery, City of Hope National Medical Center, 1500 East Duarte Road, Duarte, CA 91010 USA; 3 Department of Pathology, City of Hope National Medical Center, 1500 East Duarte Road, Duarte, CA 91010 USA

**Keywords:** Breast cancer, Surgical oncology

## Abstract

Gross intraoperative assessment can be used to ensure negative margins at the time of surgery. Previous studies of this technique were conducted before the introduction of consensus guidelines defining a “positive” margin. We performed a retrospective study examining the accuracy of this technique since these guidelines were published. We identified all specimens that were grossly examined at the time of breast conserving surgery from January 2014 to July 2020. Gross and final microscopic diagnoses were compared and the performance of intraoperative examination was assessed in terms of false positive and false negative rates. Logistic regression models were used to examine the effect of clinicopathologic covariates on discordance. 327 cases were reviewed. Gross exam prompted re-excision in 166 cases (61%). The rate of false negative discordance was 8.6%. In multivariate analysis, multifocality on final pathology was associated with discordance. We consider the false negative rate acceptable for routine clinical use; however, there is an ongoing need for more accurate methods for the intraoperative assessment of margins.

## Introduction

Breast conserving therapy (BCT), defined as breast conserving surgery (BCS) followed by radiation therapy, is one of the standard of care options for early stage breast cancer^1–5^. In order to minimize the chance of local recurrence, cancer must be completely excised—i.e., the surgical resection margins must be negative for cancer. Therefore, ensuring negative margins at the time of surgery is a major goal of the breast surgeon. Positive surgical resection margins following BCS are a strong risk factor for local recurrence^6,7^. A positive surgical margin requires additional surgery, with potential for increased morbidity, a less desirable cosmetic outcome, more patient anxiety, and increased cost to the healthcare system^8^. Persistently positive surgical margins may ultimately require a mastectomy.

The gold standard for assessing breast surgical margins is microscopic pathologic evaluation of the excised tissue following formalin fixation, paraffin embedding, and hematoxylin and eosin (H&E) staining^9^. Prior to sectioning, the margins of the specimen are typically inked in different colors so that they can be identified microscopically. This is a labor- and time-intensive process that cannot be completed intraoperatively. Therefore, any positive margins identified require a second surgical procedure. A method for rapidly and accurately identifying positive surgical margins intraoperatively would allow the surgeon to immediately excise additional tissue to achieve negative margins, thereby sparing the patient a future second surgery.

Several such methods have been proposed and investigated. The most widely used appear to be gross examination, frozen section, and imprint cytology (“touch prep”). Gross examination entails visual examination and/or palpation of a freshly excised surgical specimen without microscopic evaluation^10–12^. Frozen section analysis involves rapidly freezing tissue without formalin fixation, followed by thin sectioning and H&E staining^13–17^. Imprint cytology is performed by touching a glass slide to freshly excised tissue; cancer cells adhere to the slide, which can be immediately stained and interpreted^18–21^. Gross examination can be performed either by a pathologist or directly by the surgeon in the operating room; the other two techniques are typically performed by a pathologist.

Each of these techniques has been claimed to reduce positive margin rates, but concerns persist regarding accuracy, methodologic issues, and the need for pathologists with specific expertise (e.g. cytopathology)^22^. The College of American Pathologists (CAP) recently surveyed 866 laboratories regarding their evaluation of breast specimen margin status^23^. 265 (33%) of respondents reported performing intraoperative assessment of breast specimens; of those, 171 (66%) reported performing gross examination only, while 73 (28%) performed frozen section only, 3 (1%) performed touch prep only, and 12% used some combination of techniques.

Although gross examination seems to be the most commonly used intraoperative method, relatively few studies have analyzed the accuracy of this technique, and there are differing results^10,11^. In addition, most studies were performed prior to 2014, when there was no consensus definition for what constituted a “positive” margin^24^. Thus, these studies were forced to use arbitrary definitions which limited the generalizability of their findings (e.g., in the Balch study^11^, tumor within 2 mm of a margin was defined as “positive”, while in the Fleming study^10^, a distance of less than 10 mm was considered “positive”). In 2014, the Society of Surgical Oncology (SSO) and the American Society for Radiation Oncology (ASTRO) issued joint consensus guidelines for breast conserving surgery which established *tumor touching ink* as the definitive definition of a positive margin for invasive ductal carcinoma^25^. To our knowledge, there has been no study revisiting the accuracy of gross intraoperative assessment since the SSO-ASTRO guidelines were published.

At our institution, gross intraoperative examination by a pathologist is routinely requested for all breast conserving surgery specimens with a pre-operative diagnosis of invasive carcinoma. In this study, the primary aim was to assess the accuracy of this technique in light of the 2014 SSO-ASTRO margin guidelines. We therefore assessed the performance of the gross intraoperative method in terms of false positive and false negative rates, and analyzed clinico-pathologic variables associated with discrepancy between gross and final microscopic margin status.

## Results

During the study period, gross intraoperative margin assessment was requested for 405 patients undergoing BCS with a pre-operative diagnosis of invasive carcinoma. Only 5 patients were identified for whom no gross assessment was requested—all these had small tumors which were well visualized on pre-operative imaging. After excluding neoadjuvant treated patients, and patients for whom a quantitative gross margin distance was not provided by the interpreting pathologist, our final cohort comprised 327 patients.

**Figure 1:**
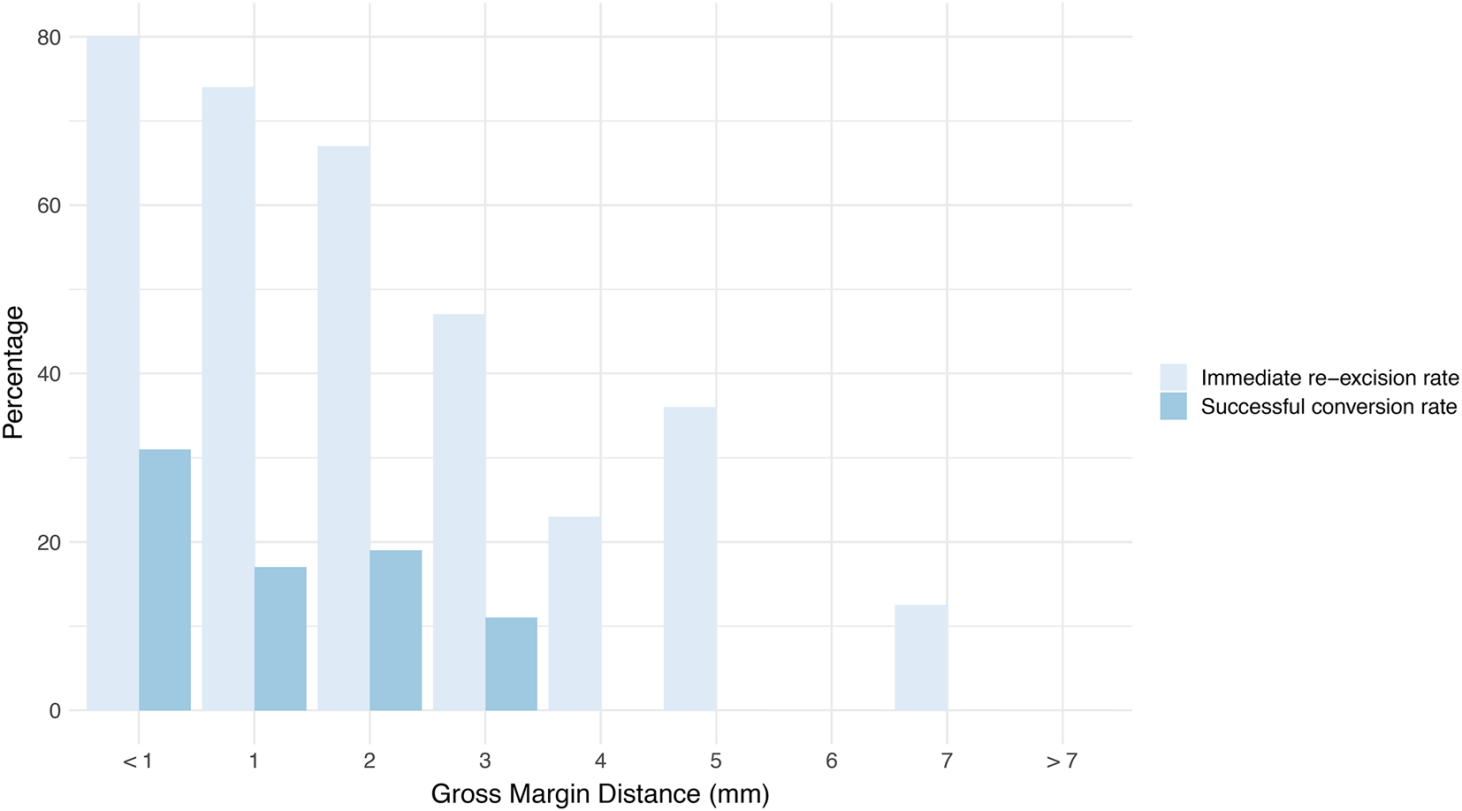
Re-excision rate and successful conversion rate by gross intraoperative margin distance. The rate of immediate re-excision (light blue) is displayed as a function of gross intraoperative margin distance. Also shown (dark blue) is the successful conversion rate as a function of gross intraoperative margin distance. For gross margin distances less than 4 mm, some patients were successfully converted to negative margin status due to gross intraoperative assessment. For gross margin distances of 4 mm or more, no re-excisions resulted in a successful conversion.

The intraoperative assessment prompted immediate re-excision in 166 cases (51% of the total cohort). Of these 166 cases, 27 (16%) represented patients whose margin status was successfully converted from positive to negative as a result of intraoperative margin assessment. These 27 patients comprised 8.3% of the total cohort, and correspond to the true positive number in Table 1. For 138 (83%) of patients who underwent re-excision, the final pathology indicated negative margins *without* considering any additionally excised tissue. These included 38 patients who were deemed to have positive margins grossly (the false positive number in Table 1). Figure 1 displays the immediate re-excision rate and the successful conversion rate as a function of the reported gross margin distance.

**Table 1:** Confusion matrix for gross intraoperative diagnosis versus final microscopic diagnosis.

Final microscopic diagnosis	Gross intraoperative diagnosis	
	Negative (262)	Positive (65)
Nsegative (272)	TN = 234 (71.6%)	FP = 38 (11.6%)
Positive (55)	FN = 28 (8.6%)	TP = 27 (8.3%)

The false negative rate was 8.6%, and the false positive rate was 11.6%. The overall test performance metrics were as follows: accuracy 80%, sensitivity 49%, specificity 86%, positive predictive value 42%, negative predictive value 89% (Table 1). In a multivariate logistic regression model, multifocality on final pathology was associated with false negative discordance (odds ratio 3.5, p = 0.02, 95% CI 1.3–9.9) (Table 2).

**Table 2:** Logistic regression model results. P-values < 0.5 are highlighted in bold.

Variable	Odds ratio	p-value	95% CI
**False negative discordance model**			
Tumor size	1.1	0.42	0.84–1.5
Tumor histologic type			
Ductal (reference)			
Lobular	1.6	0.52	0.4–7.1
Tumor grade			
1 (reference)			
2	1.0	0.99	0.3–3.4
3	1.1	0.90	0.3–4.7
Multifocal			
No (reference)			
Yes	3.5	**0.02**	1.3–9.9
Lymphovascular invasion			
No (reference)			
Yes	0.6	0.53	0.2–2.6
Lymph node stage			
0 (reference)			
1mi	0.9	0.95	0.1–8.6
1a	2.1	0.15	0.8–5.6
2a	2.4	0.40	0.3–17.3
Estrogen receptor status			
Positive (reference)			
Negative	0.4	0.48	0.04–4.4
Progesterone receptor status			
Positive (reference)			
Negative	0.9	0.86	0.2–3.2
HER2 status			
Negative (reference)			
Equivocal	0.9	0.93	0.1–7.5
Positive	0.4	0.47	0.05–3.9
Patient age > 50			
No (reference)			
Yes	0.46	0.1	0.2–1.1

## Discussion

To our knowledge, this is the only study examining gross intraoperative evaluation since the 2014 publication of the SSO-ASTRO margin guidelines. In contrast to several earlier studies, our data indicate that the technique is associated with a relatively low false negative rate of 8.6%. In other words, if a margin is deemed grossly negative, it is likely to also be microscopically negative. We therefore believe the technique is clinically useful, and can spare patients future additional surgery to achieve clear margins.

In order to optimize cosmesis, breast surgeons are usually interested in removing the minimal amount of tissue required to achieve clear margins. It would therefore be useful to establish a gross margin distance at which re-excision can be confidently avoided. At our institution, there is no agreed upon standard, and the decision to re-excise is left to the judgement of the individual surgeon. Other institutions do appear to implement a universal standard—e.g. in the study by Bolger et al.^26^, re-excision was performed for gross margin distances < 5 mm. As seen in Fig. 1, in our cohort the re-excision rate remained > 50% up to a gross margin distance of 4 mm. At a gross margin distance of 4 mm, the re-excision rate fell to approximately 25%. Importantly, re-excising for gross margin distances ≥ 4 mm did not result in *any* successful conversions. These data suggest that if the gross margin distance is at least 4 mm, re-excision can be avoided in order to optimize cosmesis without risking positive margins.

Are there any clinicopathologic variables that affect the accuracy of gross intraoperative assessment? In particular, are there any variables collected pre-operatively which could guide the surgeon when requesting gross assessment? In our multivariate logistic regression model (Table 2) only multifocality on final pathology was associated with a false negative intraoperative assessment. Multifocality is often (but not always) appreciated on pre-operative imaging and or/clinical exam; our results suggest caution when requesting a gross margin assessment in the setting of a suspected multifocal tumor. Somewhat surprisingly, no other tumor characteristics had a significant effect on the performance of gross assessment (e.g. lobular histology, tumor markers). We did not incorporate pre-operative imaging features into our model; intuitively, such features may affect the results of gross assessment, and would be interesting to explore in a future study.

In our practice, pathologists perform the gross exam and communicate the results to the surgeon. This appears to be a relatively common practice per the CAP survey cited above^23^. However, the surgeon could perform the procedure themselves, or could examine the specimen in conjunction with the pathologist, as was done in the Balch study^11^. One would expect that incorporating the surgeon’s judgement would improve the accuracy of the method, but as far as we are aware, there is no study addressing this question.

Previous studies examining the accuracy of gross intraoperative margin assessment have shown varying results. For example, a 2005 study by Balch et al.^11^ analyzed concordance between gross intraoperative margin status and final microscopic margin status for 254 consecutive patients undergoing breast conserving surgery for carcinoma (both in situ and invasive). 25% of patients ultimately underwent a second surgical procedure for margin clearance despite the use of gross intraoperative assessment. The authors concluded that the technique was not sufficiently accurate, and that other methods were needed. In the 2004 study by Fleming et al.^10^, 220 patients undergoing BCS underwent gross intraoperative margin assessment. The technique reduced the rate of future re-operation from 21.4% to 9.1%, and was associated with a low false negative rate of 3%. The authors concluded that the technique was effective. A 2011 study by Uecker et al.^12^ examined intraoperative assessment from a cost saving perspective, and found that the technique decreased total surgical costs as well as the rate of re-operation. A relatively small number of patients (17) underwent intraoperative assessment, and a “positive margin” definition was not provided.

These studies were all conducted prior to 2014, before the SSO-ASTRO consensus guidelines^25^ on margins were published. Therefore, different definitions of “positive margin” were used, and it is difficult to generalize the findings. The publication of the consensus guidelines appears to have markedly decreased the rate of re-excision for positive margins^27–30^, and has thereby somewhat decreased the necessity of an intraoperative margin assessment technique. However, a positive margin rate of 10–15% still represents a significant portion of patients who will require a second surgery. Given the high cost in terms of patient distress, potential morbidity, cosmesis, and financial burden, we still believe a rapid and accurate intraoperative tool for margin analysis is needed.

Besides the well-established pathology-assisted intraoperative methods discussed above, various radiographic and surgical tools and techniques have been shown to reduce positive margins^22^. These include pre-operative localization of the lesion via wires or newer localization devices, as well as intraoperative ultrasound. Cavity shave margin excision, in which the surgeon “shaves” an additional thin rim of tissue from the excision cavity, has also been shown to reduce positive margin rates. In a randomized clinical trial by Chagpar et al., this technique reduced positive margins by nearly 50% without an apparent adverse impact on cosmesis^31–33^.

Several novel techniques have been proposed for margin assessment, including Raman spectroscopy^34,35^, optical coherence tomography^36,37^, and confocal and multiphoton microscopy^38,39^. The MarginProbe (Dune Medical Devices, Alpharetta, GA) is a commercially available spectroscopy-based device that has been shown to reduce positive margin rates^40–42^. The Lumicell (Lumicell, Inc., Newton, MA) is a fluorescent protease probe-based system that is currently undergoing evaluation for cancer detection^43,44^. Other fluorescence-based techniques have also been described—one based on γ-glutamyl hydroxymethyl rhodamine green appears promising^45^.

Since our data was collected after the adoption of the SSO-ASTRO margin consensus guidelines, we believe our results are more generalizable than previous studies. However, we have not controlled for institution-specific surgical or pathology-related practices (e.g., the use of intraoperative specimen radiograph), and our findings should be interpreted and applied with reference to the workflow outlined above. In addition, while our cohort of 327 patients is one of the largest amongst the existing studies, a significantly larger cohort (perhaps from multiple institutions) would be ideal for bolstering our conclusions.

To conclude, in the era of consensus margin guidelines for BCS which define a “positive” margin as *no tumor touching ink*, gross intraoperative assessment appears to be a useful tool. In our cohort of 327 patients, 27 (8.3%) were spared a second surgery because of intraoperative assessment. The technique was associated with a false negative rate of 8.6%, which we believe is acceptable. Our data suggest that for gross margin distances of at least 4 mm, re-excision can safely be avoided in order to optimize cosmesis. To our knowledge, this is the only study of the gross intraoperative technique performed since 2014, and thus contributes substantially to the relatively scant existing literature.

While the SSO-ASTRO guidelines have significantly decreased the re-excision rate in BCS, the problem of positive margins persists. We anticipate and encourage the establishment of more evidence-based best practice guidelines for BCS, including pathologic assessment, in order to alleviate the persistent problem of positive margins and create more uniform care. Ultimately some of the novel techniques outlined above may play a role.

## Methods

We retrospectively identified all patients at our institution who underwent breast conserving surgery for a preoperative diagnosis of invasive carcinoma (with or without accompanying in situ carcinoma), and for whom a gross intraoperative examination was requested at the time of surgery. Excisions performed only for ductal carcinoma in situ (DCIS) were excluded, since margin guidelines differ for DCIS^46^ and intraoperative assessment is not routinely requested at our institution for these specimens. The study period was January 2014 through July 2020; the starting date was chosen to allow sufficient time for the SSO-ASTRO guidelines to be implemented by all our breast surgeons.

Specimens from 405 patients underwent gross intraoperative evaluation during the study period. For 23 specimens, a margin distance was not provided by the interpreting pathologist; rather, wording such as “tumor close to anterior margin” was used instead of a precise measurement. These specimens were excluded from the analysis. There were 55 patients who had received neoadjuvant chemotherapy (NAC) prior to gross examination. Surgeons at our institution less commonly request gross examination for NAC-treated cases since the treated tumor mass can be difficult to grossly identify in cases with a complete or near-complete pathologic response. Additionally, NAC-treated patients are likely to differ from non-NAC patients in terms of clinico-pathologic characteristics. These 55 patients were therefore excluded from the main analysis in an effort to achieve a more homogenous cohort. The clinico-pathologic features of these 55 patients are separately presented in Table S1. The final cohort comprised 327 patients.

Target margin distances for BCT at our institution follow the 2014 SSO-ASTRO guidelines^46,47^—e.g. 2 mm for ductal carcinoma in situ, and *no tumor touching ink* for invasive carcinoma. Use of intraoperative ultrasonography is surgeon-dependent and is generally used selectively. Sentinel lymph node biopsy is performed in the clinically negative axilla, and frozen sections of sentinel lymph nodes are not performed in the setting of breast conserving surgery. Many patients with clinically positive axillary nodes receive neoadjuvant systemic therapy.

Per our standard workflow, specimen radiographs with two views were obtained and interpreted by the breast surgeon and a radiologist. In our practice, these radiographs serve primarily to confirm excision of any biopsy localization devices and/or localizing wires. They are correlated with the gross intraoperative examination findings, but the latter method is generally used to assess margin status since it is assumed to be more sensitive. Occasionally, surgeons will excise additional tissue based only on the specimen radiograph findings (e.g. if the tumor mass is well visualized and appears to abut a margin). However, radiographic margin assessment is not routinely performed.

**Figure 2:**
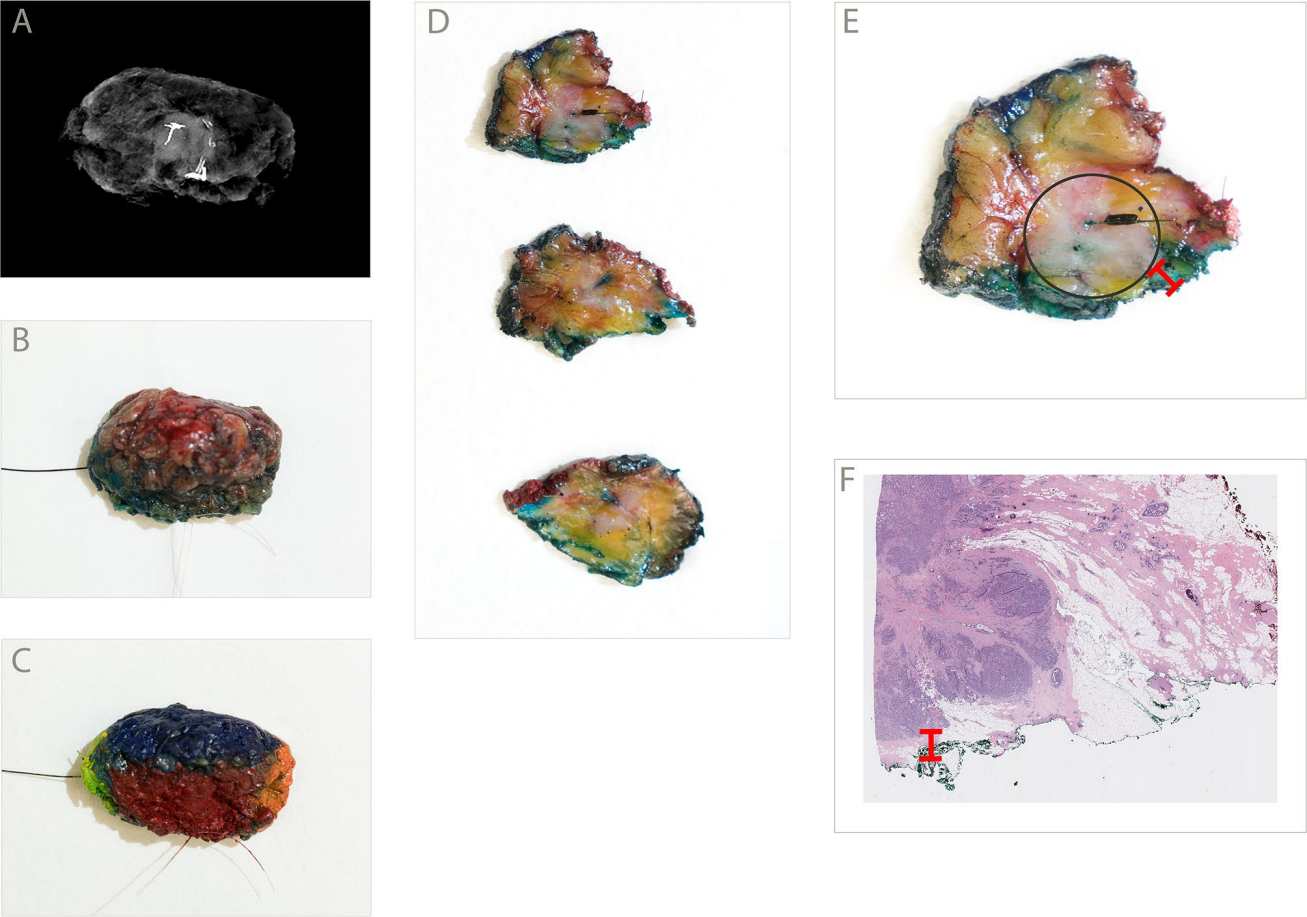
Gross intraoperative assessment workflow with example specimen. (**A**) Radiograph of a freshly excised breast lumpectomy specimen. (**B**) Excised specimen with orienting sutures and a localization wire. Dye from the sentinel node localization procedure is present. (**C**) The specimen has been inked in six colors to designate the surgical margins (inferior, superior, anterior, posterior, medial, lateral). (**D**) Representative serial sections. A centrally located tumor is visible as a vaguely defined area of whitish discoloration. (**E**) A close-up with the region of tumor annotated (black circle), as well as the grossly identified closest margin (red ruler). In this case the green-inked inferior margin was closest, and grossly measured 1 mm to the tumor. A SAVI SCOUT localization device is present (Cianna Medical, Inc.). (**F**) Microscopic pathology showing invasive ductal carcinoma. The microscopic distance to the inferior margin was 1 mm (red ruler). Gross intraoperative assessment prompted immediate re-excision of this margin, and the re-excised margin was negative for carcinoma.

Gross examination was carried out as follows (Fig. 2): after receipt in the pathology gross lab, each specimen was first radiographed to localize any markers placed at the time of biopsy, then inked in six colors to designate margins, and finally serially sectioned perpendicular to the longest axis by a Pathologists’ Assistant (PA). The PA identified any grossly visible and/or palpable lesions, at which point the supervising pathologist was called to examine the specimen as well. The pathologist was ultimately responsible for determining the closest gross margin, which was communicated to the surgeon via telephone and was also documented via a written diagnosis. The written diagnosis was incorporated into the final pathology report. Although the exact wording of the intraoperative diagnosis was left to the discretion of the individual pathologist, in practice nearly all the diagnoses included an estimated measurement in millimeters, as well as an indication of which specific margin appeared closest (e.g. “closest gross margin: anterior, 3 mm”). For 23 specimens in our cohort, a distance to the closest margin was not recorded by the pathologist (e.g. “tumor close to anterior margin”), and these specimens were excluded from the analysis. The decision to re-excise, and the amount of tissue to remove during the re-excision, was left to the discretion of the surgeon.

The diagnosis rendered during gross assessment was compared with the final microscopic pathologic diagnosis for concordance, which was considered the gold standard. A gross intraoperative diagnosis was considered “positive” if the margin distance was recorded as 0 mm, or if equivalent language was used (this was more common, e.g. “tumor abuts anterior margin”). Otherwise, the gross diagnosis was considered negative. For all final pathology reports, preliminary margins (for the initially excised specimen) and final margins (including any additionally excised tissue) were reported. A patient was deemed to have been successfully converted from positive to negative margin status if the preliminary margin was positive, but the final margins were negative after additional tissue was excised due to gross examination findings.

Test performance metrics were computed to summarize the overall performance of the gross intraoperative assessment technique, and the following clinicopathologic variables were retrieved for each specimen: tumor histologic type, grade, stage, presence of lymphovascular invasion, tumor focality, receipt of neoadjuvant therapy, and biomarker status (ER, PR, and HER2). Tumor focality was based on the final pathologic assessment as defined in the American Joint Committee on Cancer (AJCC) staging manual, 8th edition^48^, and not a gross assessment of focality. Table 3 displays the clinicopathologic variables for our patient cohort. Multivariate logistic regression models were used to examine the impact of clinicopathologic covariates on discordance. All statistics were performed using the R software package^49^.

**Table 3:** Distribution of clinicopathologic variables (total patients = 327).

Variable	Number of patients (%)
**Tumor size**	
T1mi	5 (1.5%)
T1a	18 (5.5%)
T1b	51 (15.6%)
T1c	139 (42.5%)
T2	103 (31.5%)
T3	11 (3.4%)
**Tumor histologic type**	
Invasive ductal carcinoma	295 (90.2%)
Invasive lobular carcinoma	24 (7.3%)
Other	4 (1.2%)
**Multifocal**	
Yes	38 (11.6%)
No	289 (88.4%)
**Lymphovascular invasion**	
Yes	44 (13.5%)
No	283 (86.5%)
**Lymph node stage**	
N0	213 (65.1%)
N1mi	15 (4.6%)
N1a	65 (19.9%)
N2a	9 (2.8%)
N3a	5 (1.5%)
Unknown	20 (6.1%)
**Estrogen receptor status**	
Positive	266 (81.3%)
Negative	30 (9.2%)
Unknown	31 (9.5%)
**Progesterone receptor status**	
Positive	225 (68.8%)
Negative	71 (21.7%)
Unknown	31 (9.5%)
**HER2 status**	
Negative	227 (69.4%)
Positive	18 (5.5%)
Equivocal	13 (4%)
Unknown	28 (8.6%)
**Patient age > 50 years**	
Yes	246 (75.2%)
No	79 (24.2%)
Unknown	2 (0.6%)

The study was approved by the City of Hope institutional review board, and a waiver of informed consent was obtained (IRB #19128). All methods were carried out in accordance with relevant guidelines and regulations.

## Supplementary information

Supplementary Information.

## Data Availability

The datasets generated during and/or analyzed during the current study are available from the corresponding author on reasonable request.
