# Supplementary material for: Accuracy of gross intraoperative margin assessment for breast cancer: experience since the SSO-ASTRO margin consensus guidelines
Source: Sci Rep. 2020 Oct 15;10:17344. doi: 10.1038/s41598-020-74373-6 (PMC7567822; doi:10.1038/s41598-020-74373-6)
Supplement: Supplementary file 1 — Supplementary Information. [file 41598_2020_74373_MOESM1_ESM.docx]

Accuracy of Gross Intraoperative Margin Assessment for Breast Cancer: Experience Since the SSO-ASTRO Margin Consensus Guidelines

Running Title: Accuracy of Gross Margin Assessment

Alberto Nunez, B.S.^*^, Veronica Jones, M.D. ^†^, Katherine Schulz-Costello, D.O. ^†^, Daniel Schmolze, M.D. [^‡^](https://en.wikipedia.org/wiki/Double_dagger_(typography))^§^

City of Hope National Medical Center

1500 East Duarte Road

Duarte, CA 91010

* Beckman Research Institute

† Department of Surgery

‡ Department of Pathology

§ Corresponding author

Corresponding author email: dschmolze@coh.org

**Table S1:** Distribution of clinicopathologic variables for neoadjuvant-treated patients excluded from the main cohort (total patients = 55)

| **Variable** | **Number of patients (%)** | **p-value*** |
| --- | --- | --- |
| Tumor size  T1mi  T1a  T1b  T1c  T2  T3 | 19 (34.5%)  6 (10.9%)  3 (5.4%)  8 (14.5%)  15 (27.3%)  4 (7.3%) | **< 0.001**  0.13  0.06  **< 0.001**  0.6  0.2 |
| Tumor histologic type  Invasive ductal carcinoma  Invasive lobular carcinoma  No residual carcinoma  Other | 35 (63.6%)  1 (1.8%)  18 (32.7%)  1 (1.8%) | **< 0.001**  0.15  **< 0.001**  0.54 |
| Multifocal  Yes  No | 10 (18.2%)  45 (81.8%) | 0.19 |
| Lymphovascular invasion  Yes  No | 4 (7.3%)  51 (92.7%) | 0.27 |
| Lymph node stage  N0  N1mi  N1a  N2a  N3a  Unknown | 34 (61.8%)  1 (1.8%)  13 (23.6%)  5 (9.1%)  1 (1.8%)  1 (1.8%) | 0.65  0.49  0.59  **0.04**  1  0.33 |
| Estrogen receptor status  Positive  Negative  Unknown | 36 (65.5%)  16 (29.1%)  3 (5.5%) | **0.01**  **< 0.001**  0.45 |
| Progesterone receptor status  Positive  Negative  Unknown | 24 (43.6%)  28 (50.9%)  3 (5.5%) | **< 0.001**  **< 0.001**  0.45 |
| HER2 status  Negative  Positive  Equivocal  Unknown | 28 (50.9%)  11 (20%)  3 (5.5%)  3 (5.5%) | **0.008**  **< 0.001**  0.71  0.60 |
| Patient age > 50 years  Yes  No | 32 (58.2%)  23 (41.8%) | **0.01**  **0.008** |

* p-value is the result of a Fisher exact test comparing proportions with non-neoadjuvant treated main cohort (n=327). P-values < 0.05 are highlighted in bold.
